# Histopathological effect of hypervitaminosis D_3_ on the submandibular salivary gland of albino rats and the possible protective role of vitamin K “histological & ultrastructural study”

**DOI:** 10.1186/s12903-026-08971-7

**Published:** 2026-06-29

**Authors:** Aya Y. Abo Hashim, Heba M. Eltokhey, Gihan Sh. Hassan

**Affiliations:** 1 Faculty of Dentistry, Tanta University, El-Giesh St., Tanta, Gharbia Egypt; 2 Oral Histopathology Department, Faculty of Oral and Dental Medicine, Alsalam University, Tanta, 31511 Egypt

**Keywords:** Submandibular salivary gland, Hypervitaminosis D, Vitamin K, Alizarin red, Calcification

## Abstract

**Background:**

This study was designed to inspect the effects of vitamin D_3_ overdose on the submandibular salivary gland of Albino rats and to investigate the possible protective role of vitamin K_1_ after one month, as few studies have addressed its histopathological effects.

**Methods:**

This study was carried out on 60 adult male Albino rats, and they were divided into three groups. Group I (control): received physiological saline. Group II (vitamin D_3_ overdose): received 1 ml/kg/day cholecalciferol (200 IU) for 30 days. Group III (vitamin D_3_ + vitamin K_1_): received the same vitamin D_3_ dose plus vitamin K_1_ (15 mg/kg/day) for 30 days. Blood samples were analyzed for baseline and terminal levels of total and ionized calcium. Specimens were examined using Hematoxylin and eosin, alizarin red stain, transmission electron microscopes, and histomorphometric analysis. Statistical analysis was performed using analysis of variance, followed by a post hoc test for pairwise comparisons, and paired t-test for intra-group comparison between baseline and terminal values.

**Results:**

examination of group II revealed pronounced structural alterations, notably acinar atrophy, which was statistically validated by a significant reduction in acinar circumference (p-value = 0.024*), alongside ductal degeneration, vascular congestion, and tissue calcification as detected by alizarin red stain. These histopathological changes correlated with elevated serum calcium levels (*p*-value = 0.001**). In contrast, co-treatment with vitamin K_1_ in group III substantially mitigated these pathological effects, demonstrating preservation of acinar and ductal structures, attenuation of calcific deposits, and partial normalization of serum calcium levels (*p*-value = 0.004*).

**Conclusions:**

Chronic administration vitamin D₃ overdose triggered degenerative, inflammatory, and calcific changes in the SMG. Also, co-administration of vitamin K_1_ diminished vitamin D_3_-induced histopathological changes and hypercalcemia.

## Background

In recent years, the increasing incorporation of vitamin D₃ into nutrition and multivitamin preparations has raised concerns regarding the potential risk of toxicity [1]. This risk is further exacerbated by the inappropriate prescription of high therapeutic doses in the absence of laboratory-confirmed deficiency, as well as the widespread, unsupervised use of over-the-counter supplements without adequate medical guidance. Collectively, these practices may result in excessive vitamin D intake and subsequent intoxication [2, 3].

Vitamin D is a fat-soluble vitamin with a high propensity for accumulation in body tissues, thereby predisposing individuals to hypervitaminosis [4]. Two principal forms of vitamin D are recognized: vitamin D₂ (ergocalciferol) and vitamin D₃ (cholecalciferol), both of which are essential for maintaining physiological homeostasis [4].

Although vitamin D deficiency is a widely acknowledged public health concern, hypervitaminosis D represents one of the most severe forms of vitamin toxicity. Acute toxicity may occur with daily intakes exceeding 10,000 IU, while chronic toxicity has been documented with prolonged administration of doses greater than 4,000 IU. Excessive vitamin D intake leads to sustained hypercalcemia and ectopic calcification, adversely affecting soft tissues, renal structures, cardiovascular system, and skeletal tissues [1, 5, 6]. Both experimental animal models and clinical studies in humans have consistently demonstrated pathological sequelae associated with vitamin D overexposure, including myocardial injury, vascular calcification, renal impairment, and nephrolithiasis [7–12].

Vitamin K represents another group of fat-soluble compounds, existing in four major forms (K₁, K₂, K₃, and K₄) [13]. One of the principal functions of vitamin K is preventing soft tissue calcification by modulating the activity of vitamin K-dependent proteins, including matrix Gla protein and osteocalcin [14]. Activated matrix Gla protein inhibits vascular calcification via serine phosphorylation and vitamin K-dependent γ-carboxylation of glutamate residues [15, 16].

Moreover, vitamin K facilitates osteocalcin activation by carboxylating glutamic acid residues, thereby retaining osteocalcin in the extracellular matrix and preventing its release into the surrounding tissues [17]. It is also widely distributed in body tissues, with particularly high concentrations reported in the salivary glands [18]. Within these glands, vitamin K contributes to salivary signaling and compositional regulation by mediating the carboxylation of osteocalcin and matrix Gla protein, processes dependent on calcium. Furthermore, it has been suggested that vitamin K may enhance the buffering capacity of saliva by influencing calcium and inorganic phosphate levels [19].

Noteworthy, the submandibular salivary gland (SMG) is one of the major salivary glands which is considered the principal contributor to unstimulated salivary secretion, accounting for approximately 60–65% of total saliva production [20, 21]. Despite the widespread use of vitamin D supplementation, relatively few histopathological studies have investigated the toxic effects of vitamin D on various tissues [7–12]. Moreover, few studies have addressed the effect of hypervitaminosis D_3_ and the concurrent administration of vitamin K in the context of induced hypervitaminosis D₃. Therefore, the present study aimed to assess the histopathological effects of chronic intake of vitamin D₃ overdose on the SMG of adult albino rats. Additionally, this study sought to evaluate the potential protective role of vitamin K₁ against hypervitaminosis D₃-induced histopathological alterations.

## Methods

### Animal model

All animal procedures were approved by the Ethical Committee of Faculty of Dentistry, Tanta University (#R-OB-5–24–2197) and were conducted in accordance with the guidelines laid down by the ARRIVE (Animal Research: Reporting In Vivo Experiments) guidelines for conducting animal research. The present study was conducted on sixty adult healthy male albino rats with an average body weight of 200–250 gm. Sample size calculation was performed with G Power (version 3.1.9.4, Germany) according to Chow SC et al.*, *(2017) [22], using the following equation:

$$N=\frac{{\left({Z}_{a}\right)}^{2*} {\left(SD\right)}^{2*}10}{{\left(d\right)}^{2}}$$

N = Total sample size.

*Z*_*α*_ = Is standard normal variate and its equal 3.44.

*SD* = Standard deviation of variable.

*d* = Absolute error or precision.

**Table:** 

Z_α_	SD	D
3.44	0.81	2

The criteria used for sample size calculation were as follows:

- 95% confidence limit
- 86% power of the study

Total sample size based on the results of a previous study: "Effect of vitamin D_3_ overdose and calcium supplementation in experimental nephrolithiasis model"

$$\mathrm{T}\mathrm{o}\mathrm{t}\mathrm{a}\mathrm{l}\;\mathrm{s}\mathrm{a}\mathrm{m}\mathrm{p}\mathrm{l}\mathrm{e}\;\mathrm{s}\mathrm{i}\mathrm{z}\mathrm{e}\; \mathrm{n}=\frac{{\left(3.44\right)}^{2*}{\left(0.81\right)}^{2*} 10}{{\left(2\right)}^{2}}=19\;\mathrm{p}\mathrm{a}\mathrm{t}\mathrm{i}\mathrm{e}\mathrm{n}\mathrm{t}\mathrm{s}\;\mathrm{a}\mathrm{t} \mathrm{l}\mathrm{e}\mathrm{a}\mathrm{s}\mathrm{t}\;\mathrm{i}\mathrm{n}\; \mathrm{e}\mathrm{a}\mathrm{c}\mathrm{h}\;\mathrm{g}\mathrm{r}\mathrm{o}\mathrm{u}\mathrm{p}$$

Rats were obtained and housed at the Animal House Center in the Histology Department, Faculty of Medicine, Tanta University. The animals were housed for two weeks individually in a temperature-controlled room (22 °C) with a 12-h light–dark cycle and had free access to food and water [24].

### Chemicals

Cholecalciferol (Three 400 drops) (400 IU/0.5 ml) drops were purchased from Organix food supplements, Sadat city, for Smart Company, A.R.E. Phytomenadione (Amri-K) (10 mg/1 ml) ampoules were purchased from Amriya Pharmaceutical Industries, Alexandria, A.R.E.

### Experimental design

The animals were randomly divided into 3 groups, each group consisting of 20 rats. Group I (Control Group), in which rats received a daily dose of physiological saline solution 1 ml/kg/day whereas those of group II received 1 ml/kg/day Cholecalciferol (200 IU of vitamin D_3_) [23]. Group III, in which rats were handled as in group II, with an oral therapeutic dose of vitamin K_1_ (15 mg/kg/day ≈ 3 mg daily) [25]. All doses were given orally via orogastric intubation for 30 days.

Baseline (pre-treatment) and terminal (post-treatment) blood samples were obtained from each rats all groups, centrifuged at 3000 rounds at 4 °C for 10 min to separate the serum for assessment of serum Ca, and the obtained serum samples were kept for 24 h at 2–8 °C, and then examined for ionized Ca [25, 26].

### Animal euthanasia and samples processing

At the end of the experimental period, rats were anesthetized prior to sacrifice with ketamine chloride (Ketalar, 40 mg/kg body weight), Ketalar (par pharmaceutical companies, Inc. suffern, NY, USA). Blood samples were obtained from the orbital sinus using capillary tubes. After that, the animals were euthanized to minimize pain and discomfort. The right SMG was processed for light microscopic **(LM)** examination. The left was processed for transmission electron microscopic **(TEM)** examination.

### Histopathological study

#### Light microscopic examination

Specimens were adjusted to cut serial sections of 4–5 microns in thickness and then stained with hematoxylin and eosin and Alizarin red [27] for LM examination.

#### Transmission electron microscope

TEM examination was carried out in Faculty of Science, Alexandria University, and Faculty of Agriculture, Mansoura University. A Leica UltraCut® ultra-microtome (UK) was used to prepare both semithin and ultrathin sections then examined with a Joel TEM in Medical Research Institute [28].

### Histomorphometric measurement

H&E-stained slides of all groups were visualized in a light microscope (Leica DM500 with Leica ICC50 HD Camera system). All sections were examined with light microscope in Oral Biology Department, Faculty of Dentistry, Tanta University. The circumference of acini was analyzed for quantitative measurements using the ImageJ analysis system (ImageJ 1.48 s). The software was initially calibrated automatically to convert measurement units from pixels to actual micrometer units. Using five specimens from each group, and for each specimen, examination was carried out on five fields at 400 × magnification by blinded examiners. Records for each sample were analyzed in randomly selected microscopic fields, and the mean values were calculated [29].

### Statistical analysis

All data were recorded, tabulated, and processed using the SPSS 22 (statistical package for scientific studies) data processing software for Windows. Normality of data was checked using the Shapiro–Wilk test. All statistical analyses were performed using the analysis of variance. ANOVA test (F) was used for comparison between more than two means in quantitative data. When the result of ANOVA was significant (significant F), comparisons between each two groups were performed using post- hoc Tukey test. The paired t-test was employed for intragroup comparisons of calcium levels.

## Results

### Light microscopic results

**Fig. 1:**
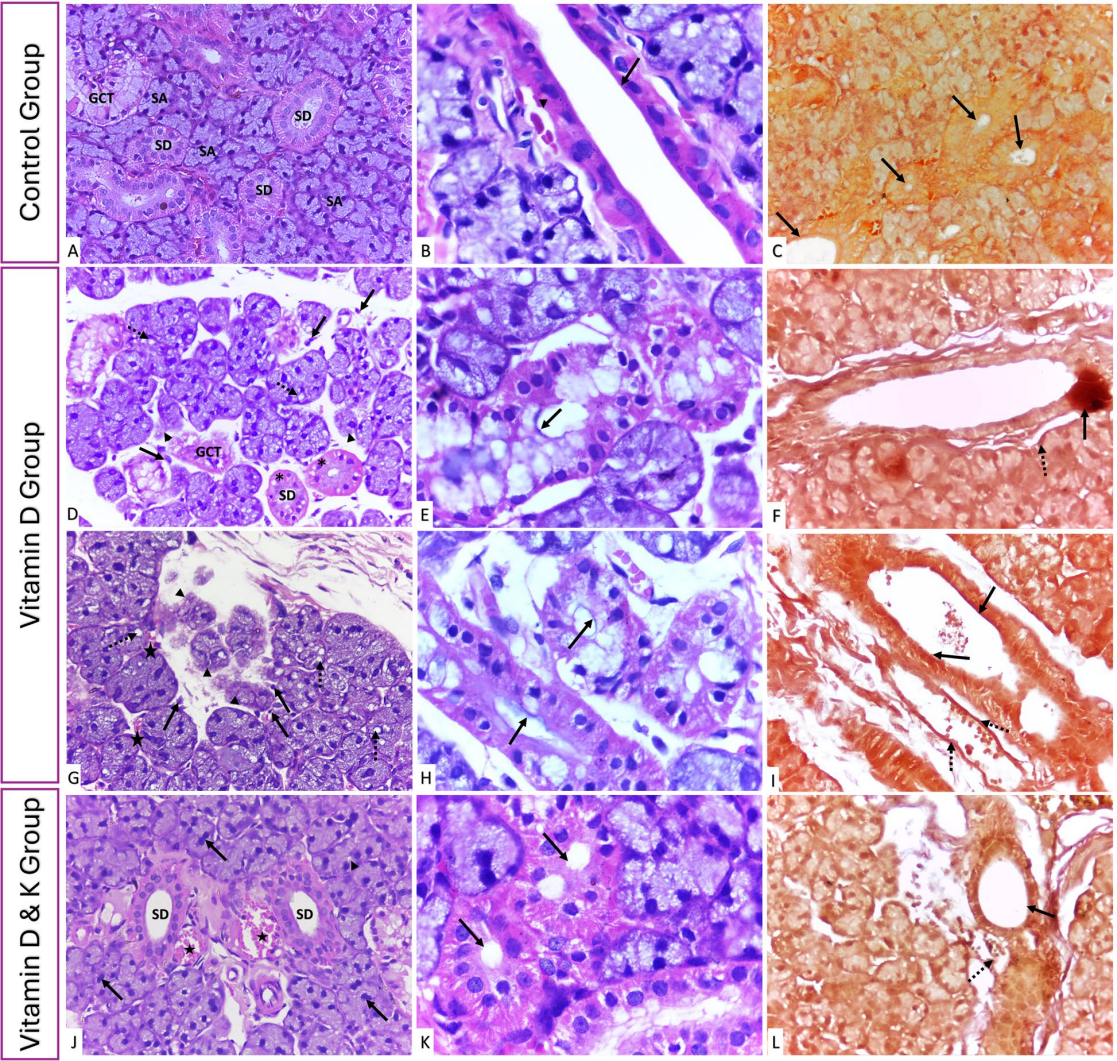
Rat SMG of control group (**A**, **B** & **C**) showing (**A**) Lobule with closely packed seromucous acini (SA). **B** squamous endothelial cells lining blood capillaries (arrowhead), and SMG duct appears normal (black arrow). **C** Red stained sections showing negative reaction in the luminal wall of the ducts (black arrows). Vitamin D group (**D**, **E**, **F**, **G**, **H** & **I**) showing (**D**&**G**) Lobules with remnants of necrotic acini (arrowheads), cytoplasmic vacuolization (dashed arrows), and inflammatory cellular infiltration (black arrows). SD with vacuolated cells and loss of basal striation (*), and complete necrosis of GCT. Congestion of blood capillaries (Star). **E** & **H** SMG ducts reveal a continuous basophilic line on their luminal side (black arrow). **F** & **I** Red stained sections showing positive reaction in the wall of the blood capillary (dashed arrows), and luminal wall of the duct (black arrows). Vitamin (D&K) group (**J**, **K** & **L**) showing (**J**) Closely packed seromucous acini with binucleated acinar cells (black arrows). Moderately congested blood capillaries (star). **K** SMG duct appears normal (black arrows). **L** Red stained sections showing decreased positive reaction in the wall of the duct (arrows) and the wall of the blood capillary (dashed arrow). (SD): striated ducts. (H&E, **A**, **D**, **G** & **J:** X40—**B**, **E**, **H** & **K**: X 100) and red stain (**C**, **F**, **I** & **L**) X 40)

SMG of vitamin D group showed peri-acinar, peri-ductal edema, and fatty degeneration in the connective tissue capsule. In addition, the acini exhibited necrotic changes, disrupted boundaries, vacuolar degeneration, and pyknotic nuclei. Moreover, vitamin D-stained sections of SMG by Alizarin red showed a positive reaction in almost all SMG, including the wall of blood capillaries and ducts. On the other hand, SMG of vitamin D&K group revealed mitigation of the structural changes induced by overdose of vitamin D_3_. Nevertheless, cytoplasmic vacuolization was still detected but decreased compared to group II. Also, there were numerous binucleated acinar cells. Additionally, red stain of vitamin D &K group showed a lesser positive reaction in all tissue sections compared to group II (Fig. 1).

### Electron microscopic results

**Fig. 2:**
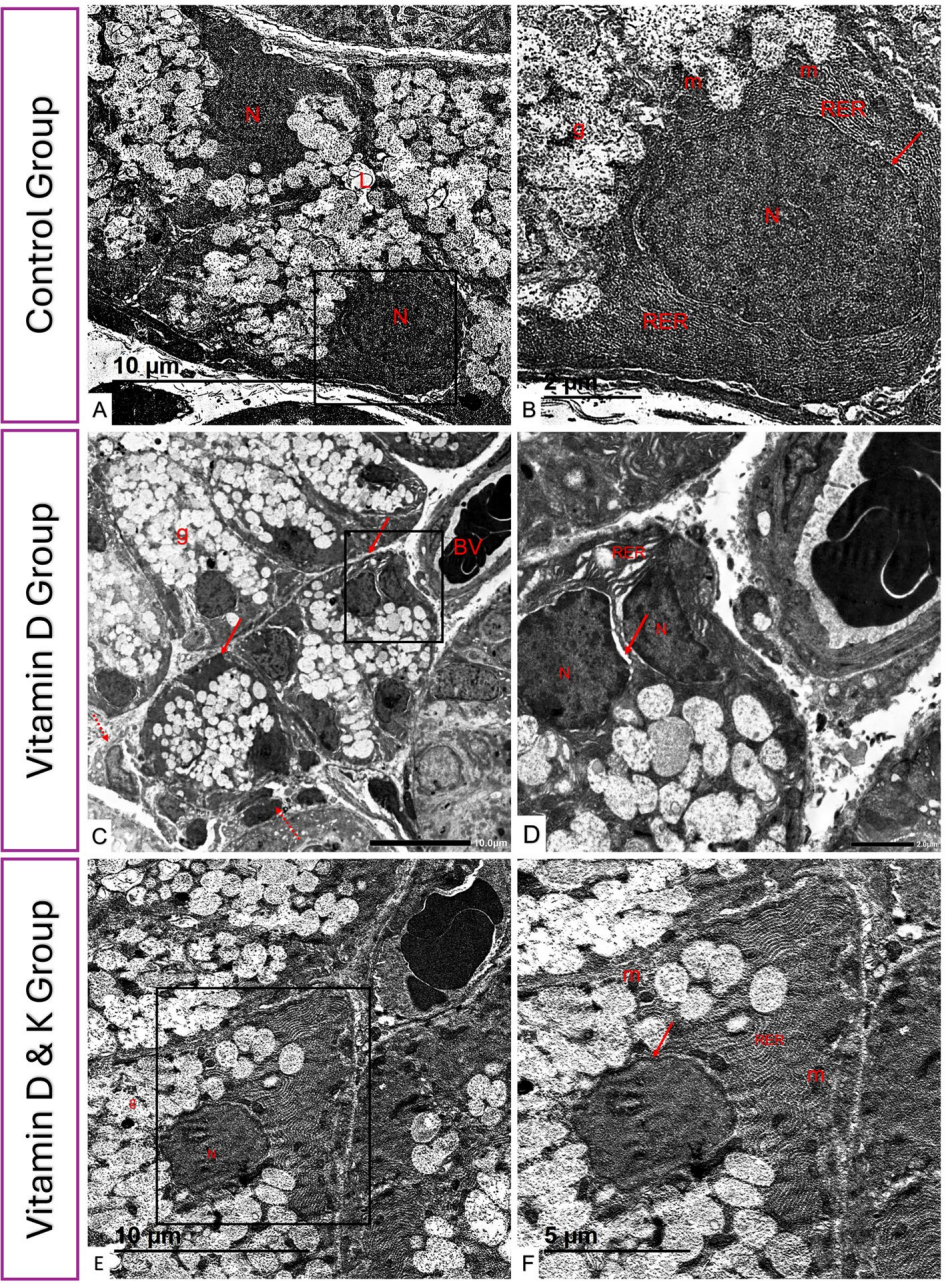
Transmission electron micrograph of serous acini of rat SMG of control group (**A**&**B**) showing (**A**) pyramidal-shaped cells surrounding a narrow lumen (L). **B** A higher magnification of the black boxed area in figure (**A**) showing a serous cell with secretory granules(g), and a basal rounded nucleus (red arrow). Serous acini of rat SMG of vitamin D group (**C**&**D**) showing (**C**) atrophied acini (red arrows), accumulated secretory granules (g), inflammatory cells infiltration (dashed arrows), and a congested blood vessel (BV). **D** A higher magnification of the red boxed area in figure (**C**) showing a nucleus with a wide perinuclear membrane (red arrow). Serous acini of rat SMG of vitamin (D&K) group (**E**&**F**) showing (**E**) serous acini with secretory granules (g), and a rounded vesicular nucleus (N). **F** A higher magnification of the black boxed area in figure (**E**) showing a slightly irregular nuclear membrane (red arrow). M: mitochondria, RER: rough endoplasmic reticulum. (Mic, orig. mag. **A**: X800, **B**: X2000), (Mic, orig. mag. **C**: X800, **D**: X2500), and (Mic, orig. mag. **E**: X800, **F**: X1200)

**Fig. 3:**
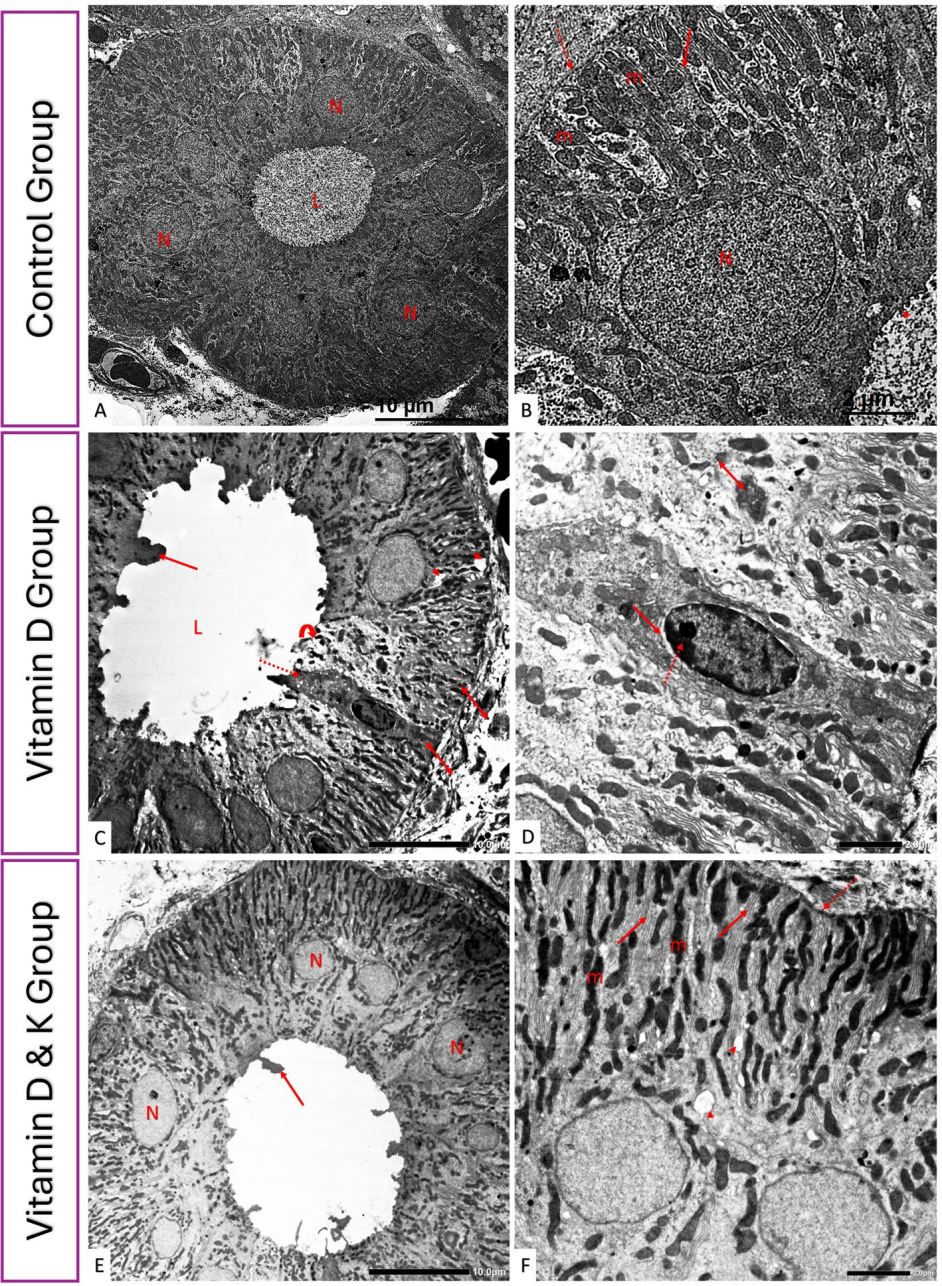
Transmission electron micrograph of striated duct of rat SMG of control group (**A**&**B**) showing (**A**) normally cells with central euchromatic nuclei (N) and a wide lumen (L). **B** A higher magnification of a normally shaped striated duct cell with a round euchromatic nucleus (N), intact basal lamina (dashed arrow), normal microvilli inside the lumen (arrowhead), basal membrane infoldings (red arrows), and normal mitochondria arranged in between these infoldings (m). Striated duct of rat SMG of vitamin D group (**C**&**D**) showing (**C**) apoptosis of the ductal cell lining (dashed arrow), necrosis (curved arrow), loss of basal striations (double headed arrows), vacuolization of ductal cells (arrowheads), and an irregular lumen border (red arrows). (**D**) A higher magnification of figure (c) showing a dark shrunken pyknotic nucleus (dashed arrow), dilated perinuclear space (red arrow) and a necrotic nucleus in the adjacent necrotic cell (double headed arrow). Striated duct of rat SMG of vitamin (D&K) group (**E**&**F**) showing (**E**) almost normal striated duct cells with large euchromatic nuclei (N) and few microvilli inside the lumen (red arrow). **F** A higher magnification of figure (**E**) showing a few vacuoles in the ductal lining (arrowheads), intact basal lamina (dashed arrow), basal membrane infoldings (red arrows), and nearly normal mitochondria arranged in between these infoldings (m). (Mic. orig. mag. **A**: X400, **B**: X1500), (Mic. orig. mag. **C**&**E**: X800, **D**&**F**: X2500)

SMG of vitamin D group showed acini with accumulated secretory granules of variable densities, wide perinuclear membranes, congested, thickened wall blood vessels, and dilated cisternae of rough endoplasmic reticulum. Moreover, the striated ducts had dark shrunken cells with dilated perinuclear spaces, loss of basal striations, vacuolization of ductal cells, and destruction of mitochondria. However, SMG of vitamin D&K group revealed partial mitigation of the changes induced by chronic overdose of vitamin D_3_. Serous acinar cells showed large coalescent secretory granules compressing the indented euchromatic nuclei, nearly normal mitochondria, and packed parallel cisternae of rough endoplasmic reticulum. Additionally, striated ducts revealed almost normal epithelial lining with large euchromatic nuclei, nearly normal RER, and elongated mitochondria arranged in between the basal membrane infoldings (Figs. 2 and 3)***.***

### Statistical results

#### Circumference of acini

The mean values of acinar circumference were measured by Image J program in the three groups. Statistical analysis of variance test revealed a significant difference in "Group I vs. Group II: p = 0.024". Also, it revealed a significant difference in "Group II vs. Group III: p = 0.001" and "Group I vs. Group III: p = 0.001" (Table 1). The greatest mean average of acinar circumference was recorded in group III, with the least value obtained in group II. Moreover, the acinar circumference in group III, in comparison with group II, increased.

**Table 1:** Descriptive and comparative statistics of acinar circumference among study groups

Circumference of acini (µm)	Group I	Group II		Group III
Range	89.80–121.59	85.65–119.04		105.60–137.81
Mean ± SD	109.1 ± 9.16	101.77 ± 10.93		124.25 ± 9.82
F. test	26.297			
*p*. value	0.001**			
Group I & Group II	Group I & Group III		Group II & Group III	
0.024*	0.001**		0.001**	

^*^ Significant *p* value < 0.05

^**^ Highly significant *p* value ≤ 0.001

#### Total and ionized Ca level

The total and ionized Ca levels were analyzed. The paired t-test was employed to evaluate the changes in baseline and terminal calcium levels within each group. In the control (Group I), no significant differences were observed in either total (p = 0.771) or ionized (p = 0.187) calcium levels (Table 2). In contrast, Group II showed a highly significant elevation in both total and ionized calcium levels (P = 0.001). Group III also demonstrated a significant increase in total calcium (P = 0.001) and ionized calcium (P = 0.004) post-treatment (Table 2).

**Table 2:** Intra-group comparison of total and ionized calcium (Ca) levels before and after treatment

Total Ca		Pre (baseline)	Post (terminal)	t. test	p. value
Group I	Range	9.27–10.6	9.38–10.41	0.294	0.771
	Mean ± SD	9.83 ± 0.45	9.79 ± 0.37		
Group II	Range	9.2–10.8	11.4–12.8	11.965	0.001**
	Mean ± SD	9.72 ± 0.63	12.06 ± 0.60		
Group III	Range	9.3–10.7	10.2–11	4.948	0.001**
	Mean ± SD	10.01 ± 0.52	10.66 ± 0.27		

Ionized Ca		Pre (baseline)	Post (terminal)	t. test	*p*. value
Group I	Range	3.82–4.32	3.87–4.34	1.344	0.187
	Mean ± SD	4.03 ± 0.15	4.09 ± 0.15		
Group II	Range	3.8–4.37	4.63–5.63	10.540	0.001**
	Mean ± SD	4.03 ± 0.18	5.01 ± 0.37		
Group III	Range	3.7–4.41	4.01–4.82	3.103	0.004*
	Mean ± SD	4.13 ± 0.22	4.37 ± 0.27		

*t* paired t- test

^*^ Significant *p* value < 0.05

^**^ Highly significant *p* value ≤ 0.001

The One-way ANOVA results revealed highly significant differences in post-treatment among the three groups (p = 0.001). The highest mean levels for both total and ionized Ca level were recorded in group II (12.06 ± 0.60 and 5.01 ± 0.37, respectively), while the lowest values were observed in group I (9.79 ± 0.37 and 4.09 ± 0.15, respectively) (Table 3). The results of post-hoc tests showed that there was a significant difference in total and ionized Ca levels between different groups. Group II exhibited a highly significant increase in both total and ionized Ca levels compared to both group I and group III (p = 0.001) (Table 3).

**Table 3:** Descriptive and comparative statistics of the mean total and ionized Ca levels between the experimental groups

Total Ca (Post)	Group I	Group II		Group III
Range	9.38–10.41	11.4–12.8		10.2–11
Mean ± SD	9.79 ± 0.37	12.06 ± 0.60		10.66 ± 0.27
F. test	136.852			
*p*. value	0.001**			
Group I & Group II	Group I & Group III		Group II & Group III	
0.001**	0.001**		0.001**	

Ionized Ca (Post)	Group I	Group II		Group III
Range	3.87–4.34	4.63–5.63		4.01–4.82
Mean ± SD	4.09 ± 0.15	5.01 ± 0.37		4.37 ± 0.27
F. test	57.262			
*p*. value	0.001**			
Group I & Group II	Group I & Group III		Group II & Group III	
0.001**	0.003*		0.001**	

*F* ANOVA test

^*^ Significant *p* value < 0.05

^**^ Highly significant *p* value ≤ 0.001

## Discussion

Hypervitaminosis may develop gradually with chronic exposure or occur acutely following excessive intake in a single episode. Hypervitaminosis is particularly associated with fat-soluble vitamins, such as vitamin D, owing to their tendency to accumulate in body tissues. Hypervitaminosis D is frequently iatrogenic, resulting from the prescription of high doses without definitive confirmation of vitamin D deficiency [4]. Moreover, several chronic bone disorders (e.g., osteomalacia and osteoporosis) and autoimmune diseases (e.g., rheumatoid arthritis, systemic lupus erythematosus, and psoriasis) are commonly associated with reduced circulating levels of vitamin D metabolites. Consequently, prolonged administration of high daily doses of vitamin D₃ is often implemented, which increases the risk of hypervitaminosis [30]. Also, in the present study, the submandibular gland was selected due to its relatively large size and ease of dissection in rodent model [31]. Furthermore, the SMG constitutes the principal contributor to unstimulated salivary secretion, accounting for approximately 60–65% of total saliva production. Notably, the SMGs of rats exhibit considerable anatomical and functional similarities to those of humans [20, 21]. To date, no studies have specifically investigated the effects of vitamin D overdose on the SMG. Accordingly, the present study aimed to evaluate the effects of chronic vitamin D₃ overdose on the SMG and to investigate the potential ameliorative role of vitamin K. The selection of rats weighing 200–250 g corresponds to the young adult stage, at which physiological systems—particularly calcium–phosphate homeostasis, renal function, and vitamin D metabolism—are fully matured and functionally stable [32].

Remarkably, LM & TEM examination of Group II demonstrated marked histopathological alterations reflecting the destructive effects of chronic hypervitaminosis D_3_ on SMG architecture. Cytoplasmic vacuolization in acinar and ductal cells is widely regarded as an early morphological indicator of cellular degeneration and apoptosis [7, 25, 33]. This was attributed to oxidative stress mediated by reactive oxygen species, leading to lipid peroxidation, mitochondrial and rough endoplasmic reticulum damage, membrane disruption, inflammation, and apoptotic degeneration. Similar alterations were reported by Aboayana et al., (2025) in rat SMG following exposure to titanium dioxide nanoparticles [34].

Moreover, LM examination depicted atrophied and necrotic acini in group II. Such alterations could be attributed to hypercalcemia-induced extracellular matrix remodeling, mediated by transforming growth factor-β and matrix metalloproteinase activation, resulting in basement membrane degradation and disruption of acinar integrity [35].

Ultrastructurally, acinar and ductal cells exhibited pronounced nuclear and cytoplasmic degeneration. Nuclear changes included chromatin condensation, shrinkage, pyknosis, and perinuclear membrane dilation, while cytoplasmic alterations involved mitochondrial swelling with cristae disruption, fragmented and dilated RER, and extensive vacuolization. These changes are characteristic of apoptotic injury mediated by hypercalcemia and oxidative stress [36, 37]. Furthermore, excessive intracellular Ca^2^⁺ overload may exceed cellular repair capacity, shifting cell death pathways from apoptosis toward necrosis [37].

Mitochondrial degeneration within striated ducts was a prominent ultrastructural feature in group II. This damage could result from excessive free radical generation or preferential binding of toxic metabolites to mitochondrial DNA, leading to impaired ATP synthesis and mitochondrial membrane dysfunction [38, 39]. Moreover, irregular luminal borders of striated ducts were observed, consistent with findings reported by Ashraf B, et al., (2020) [40] who attributed similar changes to ROS-mediated membrane injury and inflammatory mediator activity, leading to its rupture and distortion [41].

Peri-acinar and peri-ductal edema were notable findings in group II. These changes could be attributed to hypercalcemia induced by excess vitamin D_3,_ which led to calcium deposition in alveolar and interalveolar septa of the lung [7, 25]. Hypercalcemia-induced dysregulation of Ca^2^⁺-dependent ion channels and transporters, including TMEM16A, Na⁺/K⁺-ATPase, and Cl⁻ channels, may disrupt osmotic balance and fluid transport, resulting in interstitial fluid accumulation and edema [42–44].

Connective tissue septa in group II exhibited fatty degeneration and inflammatory cell infiltration. These results were consistent with previous reports of vitamin D₃ toxicity in hepatic tissues [7, 45, 46]. Fatty degeneration could be linked to mitochondrial dysfunction secondary to calcium overload, which impairs β-oxidation and lipid metabolism [47]. Inflammatory infiltration might result from ROS-induced cellular stress and chemotactic signaling, as well as the release of damage-associated molecular patterns from necrotic cells, promoting macrophage and lymphocyte recruitment [48, 49].

Vascular calcification was a striking feature in group II and was confirmed by positive Alizarin Red staining. Similar findings have been reported in multiple organs following vitamin D₃ toxicity [25, 45, 50]. Excess vitamin D₃ could promote vascular calcification by inducing osteogenic differentiation of vascular smooth muscle cells through hypercalcemia and hyperphosphatemia [51]. Moreover, chronic inflammation could lead to vascular calcification, which is caused by activated macrophages that produce proinflammatory cytokines such as TNF-α, oncostatin M, IL-6, and IL-1β. These pro-inflammatory cytokines could encourage smooth muscle cells in blood arteries to differentiate into osteogenic tissue [52].

In contrast, group III demonstrated substantial histological and ultrastructural preservation of SMG architecture. Light and electron microscopic findings revealed preventive effects of vitamin K with less sever histopathological damage and significantly reduced calcification. These findings could be attributed to the antioxidant, anti-inflammatory, and cell proliferation–enhancing effects [53].

The protective role of vitamin K was further supported by the preserved mitochondrial integrity, reduced vacuolization, and maintained RER organization. Previous studies demonstrated that vitamin K mitigates oxidative stress, preserves mitochondrial function, and modulates endoplasmic reticulum stress pathways [54–56]. Additionally, vitamin K maintains matrix Gla protein (MGP) activity and promotes osteocalcin carboxylation, thereby preventing ectopic calcification and preserving soft tissue architecture [17, 45, 57]. Activated MGP functions as a strong calcification inhibitor because it contains three serine residues and five glutamic acid residues. Two essential post-translational modifications occur in MGP: the serine phosphorylation and the vitamin K-dependent carboxylation of Glu, which may reduce the accumulation of calcium on the inner lining of BVs [15].

Histomorphometric analysis revealed a significant reduction in acinar circumference in group II, confirming acinar atrophy, consistent with prior studies reporting organ weight reduction following chronic vitamin D₃ toxicity. They attributed this reduction to the elevation of Ca levels, which led to cellular toxicity, apoptosis, ischemia, and necrosis, resulting in loss of parenchymal cell mass and consequently reduced organ weight [58]. Conversely, group III exhibited a significant increase in acinar circumference compared to group II, reflecting prevention of atrophy induced by vitamin d overdose. The maintenance of acinar circumference agreed with Nakagawa K et al., (2019) who demonstrated that coadministration of Vitamin K maintained cell morphology, survival, and function of pancreatic acinar cells [59].

Biochemical analysis confirmed hypercalcemia in group II, with significant elevations in total and ionized calcium levels, in agreement with previous reports [7, 45]. Although group III also exhibited elevated calcium levels, these values were significantly lower than those of group II, indicating reduced calcification severity. This contradicts the results of Elshama et al., (2016)**,** who found a non-significant difference in serum Ca between control and vitamin K groups [45]. These divergent findings may be ascribed to the vitamin D intoxication regimens administered, as they used only a single toxic dose of vitamin D_3_.

## Limitation

The use of a single, potentially non-toxic dose/duration of Vitamin D3.

The preventive, rather than interventional, study design.

The lack of mechanistic data (e.g., measurements of oxidative stress, apoptosis markers, or vitamin K-dependent protein activity like carboxylated Matrix Gla Protein).

The qualitative nature of much of the histopathological assessment, aside from acinar circumference.

## Conclusions

Collectively, the present study demonstrates that chronic vitamin D₃ overdose induced degenerative, inflammatory, and calcific changes in the SMG. Also, vitamin K_1_ co-administration as a preventive model, mitigated vitamin D_3_-induced histopathological changes and hypercalcemia. Therefore, excessive and unmonitored intake of vitamin D₃ should be avoided due to its potential toxic effects on salivary gland integrity and calcium homeostasis. Further clinical and translational studies are warranted to evaluate the potential protective role of vitamin K₁ in humans exposed to elevated levels of vitamin D₃. In addition, future research should investigate different doses and durations of vitamin K₁ administration to establish optimized, safe, and evidence-based therapeutic protocols.

## Data Availability

All data are available from the corresponding author upon a reasonable request.

## Abbreviations

ANOVA: Analysis of variance
IU: International unit
IL: Interleukin
LM: Light microscope
MGP: Matrix Gla protein
RER: Rough endoplasmic reticulum
ROS: Reactive oxygen species
SMG: Submandibular salivary gland
TEM: Transmission electron microscope
TNF: Tumor necrosis factor
TMEM16A: Transmembrane member 16A
